# Polyesteracetals via TBD Catalyzed Ring‐Opening Polymerization

**DOI:** 10.1002/marc.202500273

**Published:** 2025-07-07

**Authors:** Jakob Meyer, Natalie E. Göppert, Leanne M. Stafast, Christine Weber, Ulrich S. Schubert

**Affiliations:** ^1^ Laboratory of Organic Chemistry and Macromolecular Chemistry (IOMC) Friedrich Schiller University Jena Jena Germany; ^2^ Jena Center for Soft Matter (JCSM) Friedrich Schiller University Jena Jena Germany

**Keywords:** block copolymers, organocatalysis, poly(2‐oxazoline)s, polyesteracetals, ring‐opening polymerization

## Abstract

Polyacetals represent a novel category of degradable polymers, exhibiting remarkable potential for utilization in biomedical and pharmaceutical applications due to their controllable degradation behavior under physiological conditions. The organocatalyst 1,5,7‐triazabicyclo[4.4.0]dec‐5‐ene (TBD) was used for the ring‐opening polymerization (ROP) of 2‐methyl‐1,3‐dioxane‐4‐one. Whereas standard reaction conditions, suitable for lactide polymerization resulted in a polyester due to loss of acetaldehyde, the amount of esteracetal repeating units could be tailored by the polymerization conditions. Performing the ROP at −35°C at a monomer concentration of 15 mol L^−1^ maximized the incorporation of labile acetal groups to 50 mol%. The versatility of this polymerization system was demonstrated through the successful initiation with a wide range of alcohols, including 1‐pyrenemethanol for end‐group analysis via size exclusion chromatography with UV detection and matrix‐assisted laser desorption‐ionization mass spectrometry, as well as various macroinitiators such as mono‐ and bifunctional poly(ethylene glycol)s, poly(2‐ethyl‐2‐oxazoline), poly(2‐*n*‐nonyl‐2‐oxazoline), and poly(2‐*iso*‐propyl‐2‐oxazoline), thereby facilitating the synthesis of well‐defined block copolymers.

## Graphical abstract

The ring‐opening polymerization (ROP) of a cyclic hemiacetal ester catalyzed by 1,5,7‐triazabicyclo[4.4.0]dec‐5‐ene (TBD) yields statistical copolymers containing esteracetal as well as ester repeating units. Their ratio can be tailored through adjustment of the ROP conditions. The use of hydroxyl end‐functionalized poly(ethylene glycol)s and poly(2‐oxazoline)s as macroinitiators enables access to well‐defined block copolymers.

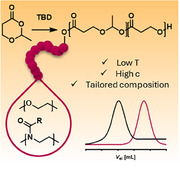

## 1 Introduction

Polyesters represent the main biodegradable polymer type used in biomedical applications and drug delivery. Prominent examples include aliphatic polyesters such as poly(*ε*‐caprolactone), poly(lactic acid), poly(lactic acid‐*co*‐glycolic acid), or poly(3‐hydroxybutyrate). However, acidification of the microenvironment may occur upon degradation as the respective hydroxy acids are formed through hydrolysis. This can cause protein instability [1, 2], accelerate degradation [3], or affect the stability of encapsulated active ingredients [4]. Polyacetals represent a class of polymers that may be able to overcome some of these challenges. The acetal moieties are stable at alkaline conditions but quickly degrade in acidic conditions forming aldehydes and alcohols [5].

The reaction of diols with divinyl ethers [6], or the transacetalization of diols with 2,2‐dimethoxypropane represent common ways to obtain polyacetals [7]. However, synthesis of polymers with well‐defined molar mass and end groups is challenging via such step‐growth approaches. The cationic copolymerization of carbonyl compounds with vinyl ethers developed by Aoshima and coworkers is one way to obtain a variety of polyacetals in a chain‐growth polymerization [8]. Also the common poly(oxymethylene) represents a polyacetal, which is obtained via rin‐g‐opening polymerization (ROP) of trioxane.

Hemiacetalesters are heterocyclic compounds that yield polyesteracetals through ROP, as demonstrated by Neitzel et al., who polymerized 2‐methyl‐1,3‐dioxan‐4‐one (**MDO**) using ZnEt_2_ as catalyst in low amounts [9]. However, loss of the carbonyl compound may occur during the ROP, thereby producing a polyester.

In addition, diphenyl phosphoric acid has been successfully applied for the ROP of **MDO** [10].

A range of organobase catalysts has been developed over the past years that are ideal for the ROP of lactones, i.e., cyclic esters [11, 12, 13, 14, 15, 16]. Organobases offer the possibility to introduce defined α‐end groups to the resulting polyester through the ability to use an alcohol as initiator. It is hence surprising that such catalysts have only scarcely been investigated in the ROP of cyclic hemiacetal esters. To the best of our knowledge, only the copolymerization of lactide with 1,3‐dioxolan‐4‐one has been attempted but yielded purely polyester due to the loss of formaldehyde [17].

**SCHEME 1:**
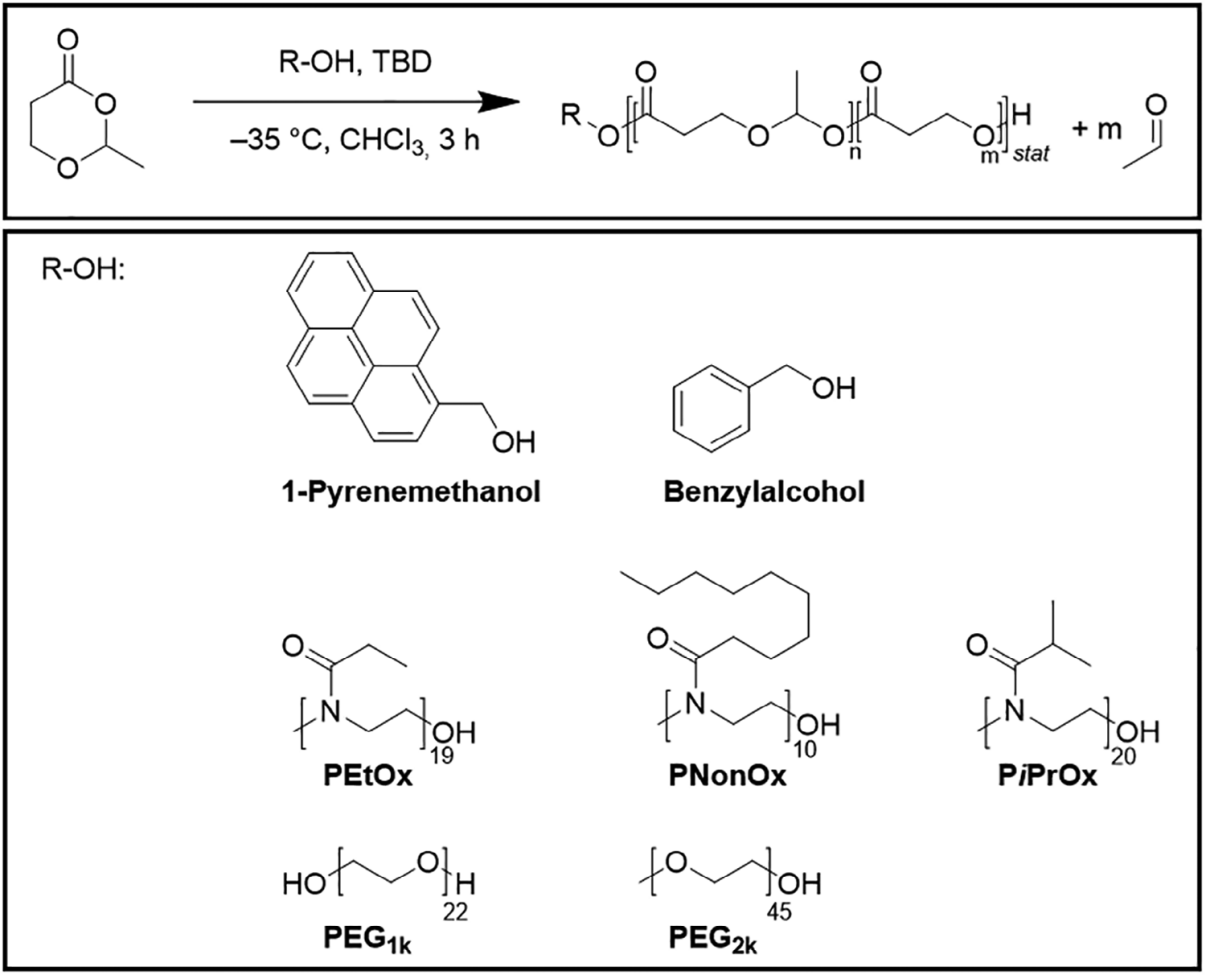
Schematic representation of the polymerization of MDO and the used initiators.

We hence explored that field using **MDO** as a monomer and widely used organobase catalyst systems such as Takemoto's catalyst [13], the combination of 1,8‐diazabicyclo(5.4.0)undec‐7‐ene (DBU) [14] and thiourea (TU) [16], and TBD [15]. In addition to benzyl alcohol and pyrenemethanol, various macroinitiators were applied to enable access to block copolymers comprising a polyesteracetal block (Scheme 1).

## 2 Results and Discussion

The monomer **MDO** was obtained via Bayer–Villiger oxidation of 2‐methyldihydrofuran‐3(2*H*)‐one with *meta*‐chloroperbenzoic acid according to Neitzel et al. (Scheme S1) [9].

### 2.1 Screening of ROP Conditions

For an initial screening of catalyst type and reaction conditions, benzyl alcohol (BnOH) was selected as the initiator at an [M]/[I] ratio of 20. Parameters such as concentration, temperature, and catalyst equivalents were altered (Table S1). Monomer conversion and the composition of the formed polymers were estimated by means of ^1^H‐NMR spectroscopy measurements of aliquots taken from the reaction solutions as shown in Figure S6.

Irrespective of the catalyst and solvent, polymerizations at room temperature at a monomer concentration of 0.7 mol L^−1^ yielded the polyester due to the loss of acetaldehyde. Also conducting the polymerization at 60°C resulted in the polyester. It seems reasonable to assume that the loss of acetaldehyde is an entropically favored process. Taking into account the Gibbs–Helmholtz equation (ΔG = ΔH – TΔS), the entropic term TΔS would contribute less to the Gibbs Energy ΔG at lowered temperature, making an elimination less favorable. As Takemoto's catalyst gave only low monomer conversions, it was excluded from further studies.

**FIGURE 1:**
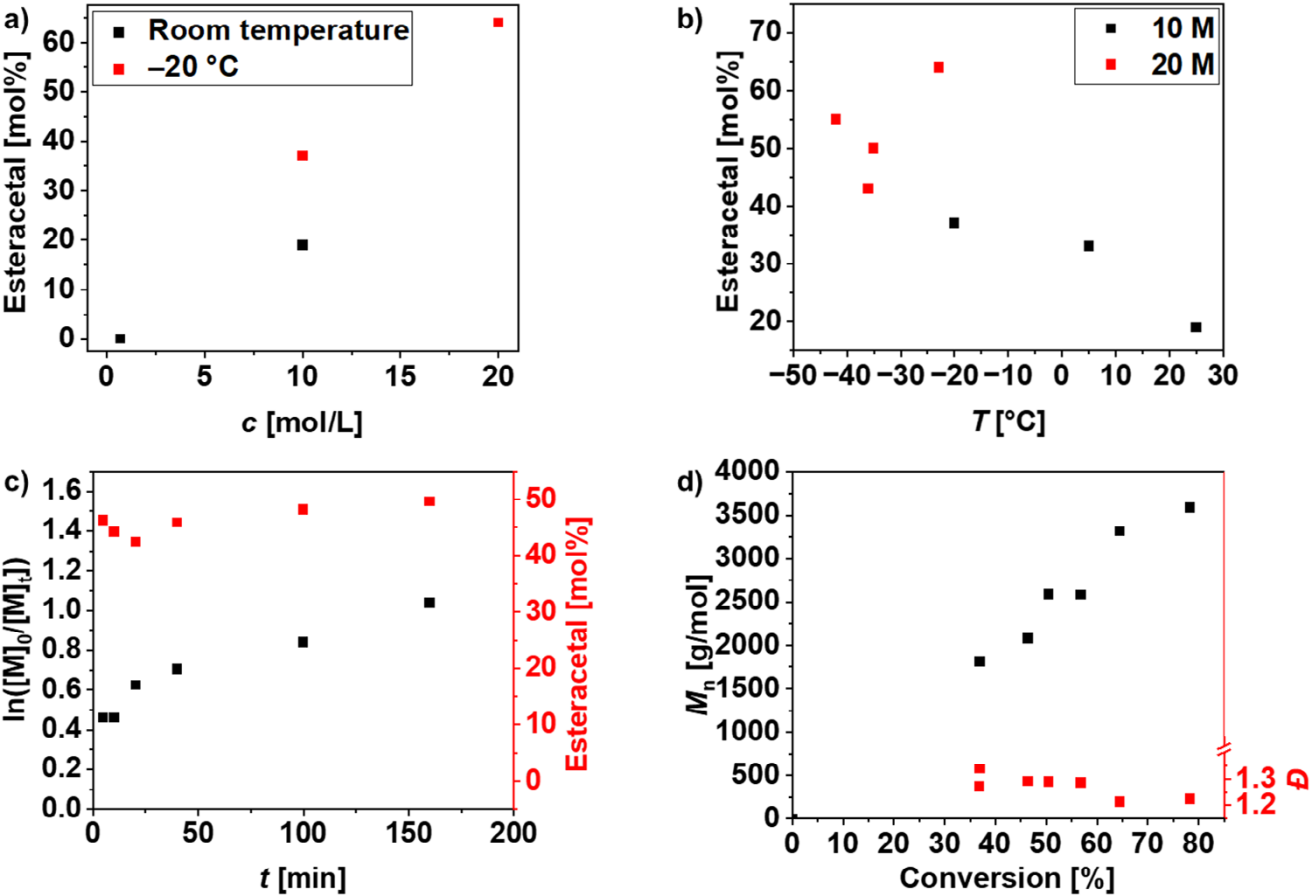
Screening of reaction conditions for the TBD catalyzed ROP of MDO. (a) Dependence of the esteracetal content on monomer concentration ([M]/[BnOH]/[TBD] = 20/1/1). (b) Dependence of the esteracetal content on reaction temperature ([M]/[BnOH]/[TBD] = 20/1/1). (c,d) Kinetic studies of the ROP of MDO with BnOH as initiator and TBD as catalyst ([MDO]/[BnOH]/[TBD] = 100/1/5) at −35°C.

Increasing the monomer concentration (10 M) yielded a statistical copolymer containing 81 mol% ester as well as 19 mol% esteracetal units. The amount of the latter was further increased to 33 mol% when the ROP was conducted at 5°C. The effects occurred with both organobase catalyst systems, i.e., DBU/TU as well as TBD, but were more pronounced when TBD was used. In consequence, TBD was selected for further optimization (Table S1). In summary, lowered reaction temperatures, increased monomer concentration and catalyst amount seemed favorable to increase the esteracetal fraction in the polymer (Figure 1).

More detailed kinetic studies were conducted at −35°C at an [M]/[BnOH]/[TBD] ratio of 100/1/5, enabling reasonable time intervals for sampling. As evident from the first‐order kinetic plot (Figure 1c), the ROP proceeded particularly fast in the initial stages, which is likely associated with the addition of the initiator and the catalyst to the monomer while maintaining the reaction temperature. However, the esteracetal content remained almost constant throughout the course of the ROP, indicating that the esteracetal moieties are evenly distributed along the formed polymer chain. As the molar mass increased in a linear fashion with monomer conversion and monomodal molar mass distributions with dispersity values below 1.3 were observed, the ROP should be controlled with respect to molar mass.

### 2.2 End Group Analyses

To investigate if the ROP also proceeded in a controlled fashion with respect to the polymer end group, BnOH‐initiated PMDO was purified by preparative size exclusion chromatography. ^1^H‐NMR spectroscopy clearly indicated the presence of the benzyl α‐end group, enabling to determine the degree of polymerization of the esteracetal and ester repeating units, which was in good agreement with the values expected from conversion and [M]/[I] ratio (Table 1). In addition, the esteracetal content was not affected by the purification procedure. Analysis by means of MALDI‐TOF MS clearly indicated the presence of both repeating units (Figure S10) as distributions spaced by *m/z* differences of 72 (ester repeating unit) as well as 116 (esteracetal repeating unit) were observed. However, end‐group determination proved difficult, possibly due to degradation or fragmentation of the polymer during the measurement.

**TABLE 1:** Selected characterization data of the polyesteracetal homo‐ and block copolymers. ROP conditions: [M]/[I]/[TBD] = 100/1/5, T = −35°C, [M]_0_ = 15 m in CHCl_3_.

	Conv. (%)	EA ^a^ Crude (mol%)	EA ^b^ Purified (mol%)	*M*_n_ (SEC) (g/mol)	*Đ* (SEC)	DP ^c^ I/EA/E
BnPMDO	46	46	44	1900	1.37	‐/26/24
PyPMDO	25	55	54	1500	1.15	‐/7/6
PEtOx‐PMDO	42	43	43	3800	1.10	19/12/16
PNonOx‐PMDO	18	33	31	3100	1.18	10/6/12
P*i*PrOx‐PMDO	17	30	22	3300	1.09	20/4/13
PMDO‐PEG‐PMDO	34	44	44	5600	1.06	23/15/19
PEG‐PMDO	27	32	37	6400	1.05	45/10/17

^a^ mol% of esteracetal in the PMDO block determined by ^1^H‐NMR spectroscopy of the reaction solution.

^b^ mol% of esteracetal in the PMDO block determined by ^1^H‐NMR spectroscopy of the purified polymer.

^c^ Degree of polymerization of macroinitiator (I), esteracetal repeating units (EA), and ester repeating units (E) in the PMDO‐containing polymers.

**FIGURE 2:**
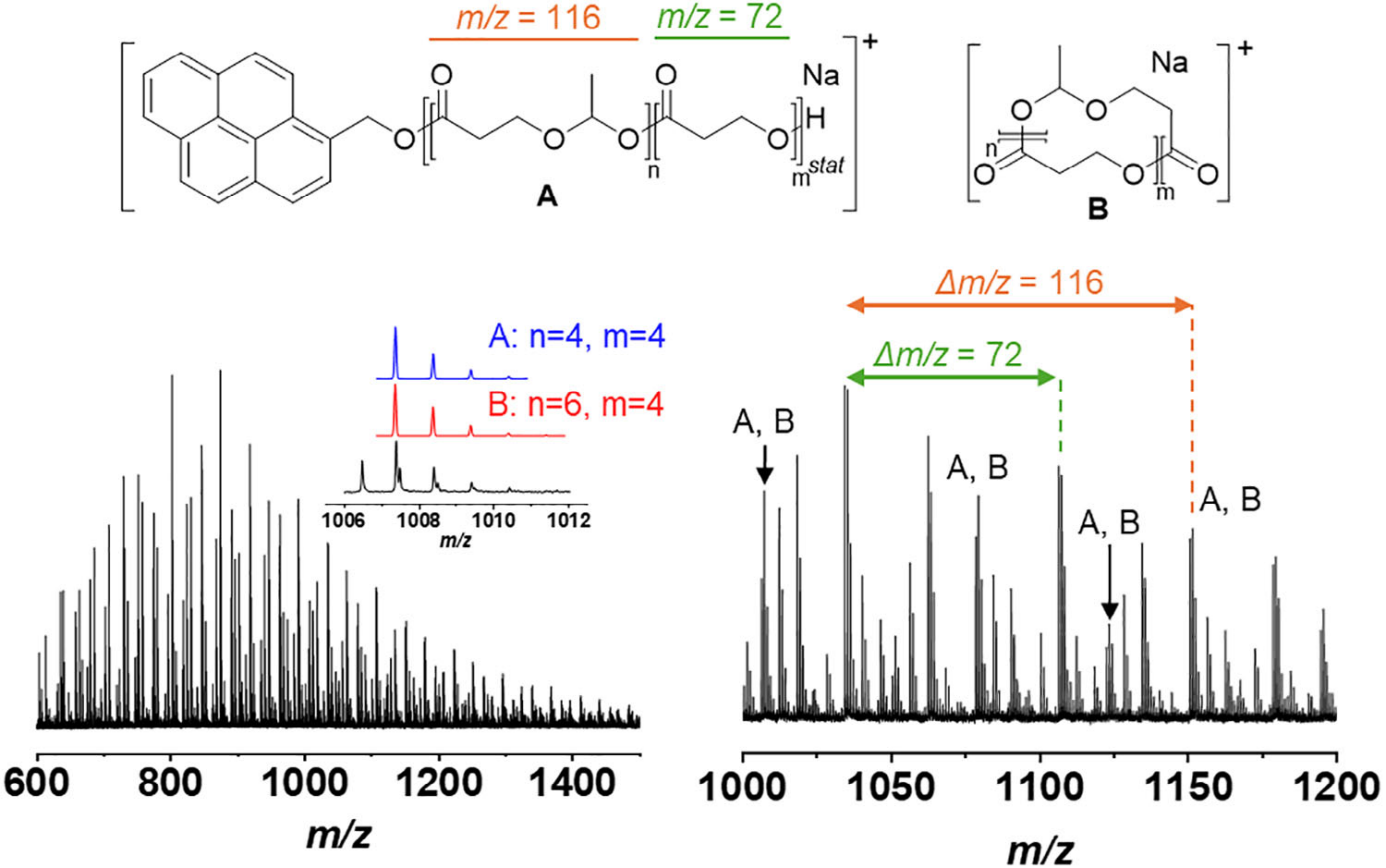
MALDI‐TOF mass spectrum of the purified PyPMDO (DCTB, NaTFA). The inserted graph shows an overlay of the measured (black) and calculated isotopic patterns of species A (blue) and B (red).

To verify if the initiator was covalently attached to the PMDO, 1‐pyrenemethanol was used to initiate the ROP. Again, MALDI‐TOF MS analysis indicated the presence of both types of repeating units (Figure 2). Comparison of measured isotopic patterns with calculated ones hinted toward the presence of the pyrene moiety as the end group of the polymer (species A in Figure 2). However, cyclic polymer species, which are a common byproduct formed by intramolecular transesterification during the ROP of lactones [18], are isobaric when the number of repeating units is adjusted (species B in Figure 2). Nevertheless, the covalent attachment of the pyrene moiety was evident from SEC analysis with UV detection at 330 nm (Figure S12).

### 2.3 Synthesis of Block Copolymers

The apparent end group control was further investigated by utilization of various macroinitiators for the ROP of **MDO** (Table 1). Two poly(ethylene glycol)s (PEG) were selected for that purpose. Featuring two hydroxyl end groups, PEG_1k_ represents a bifunctional macroinitiator to yield an BAB triblock copolymer. In contrast, the monofunctional PEG_2k_ with an ω‐terminal hydroxyl end group would enable access to a AB diblock copolymer. The range of macroinitiators for the ROP of **MDO** was further extended by three ω‐hydroxyl terminal poly(2‐oxazoline)s (POx) with altered hydrophilicity: [19, 20, 21] Poly(2‐ethyl‐2‐oxazoline) (PEtOx), poly(2‐*iso*‐propyl‐2‐oxazoline) (P*i*PrOx), and poly(2‐*n*‐nonyl‐2‐oxazoline) (PNonOx). These were synthesized by termination of the cationic ROP of the respective 2‐oxazoline monomers with triethylammonium acetate and subsequent NaOMe‐catalyzed transesterification with methanol according to a procedure well‐established for PEtOx (Scheme S2) [22, 23, 24]. According to characterization by means of SEC, ^1^H‐NMR spectroscopy, and MALDI‐TOF MS, the procedure could be transferred to the other POx almost without any changes (Figures S15–S21). Consequently, these polymers were effective macroinitiators for the polymerization of **MDO**.

**FIGURE 3:**
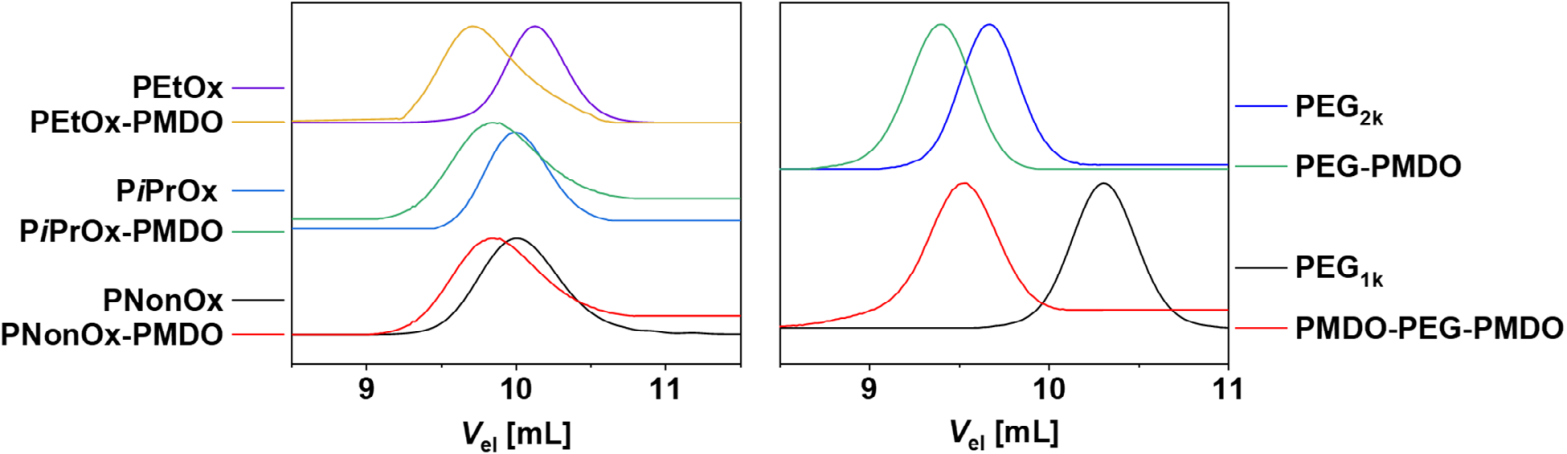
Overlay of SEC elugrams of the PMDO‐containing block copolymers with the respective macroinitiators (CHCl_3_, RI detection).

Signals assigned to PMDO as well as PEG or POx, respectively, were assigned in the ^1^H‐NMR spectra of all purified block copolymers (Figures S22–S26). In addition, clear peak shifts were observed upon the comparison of the SEC elugrams of the block copolymers and the respective macroinitiators (Figure 3). All block copolymers featured unimodal molar mass distributions with dispersity values *Ð* < 1.2. This holds true, particularly for **PMDO‐PEG‐PMDO** and confirms that block copolymers were successfully obtained. The esteracetal content in the PMDO blocks was in a similar range for all block copolymers (30–45 mol%). In summary, the α‐end group and acetal moieties were successfully retained.

## 3 Conclusion

We presented the TBD‐catalyzed ROP of the cyclic hemiacetal ester monomer 2‐methyl‐1,3‐dioxan‐4‐one (**MDO**). Whereas standard reaction conditions, suitable for lactide polymerization resulted in a polyester due to loss of acetaldehyde, the amount of esteracetal repeating units could be tailored by the polymerization conditions. It increased to around 50% through increasing the monomer concentration and lowering the reaction temperature. Various alcohols and macroinitiators were suitable to initiate the ROP, enabling access to PMDO with defined α‐end groups and block copolymers comprising POx as well as PEG with narrow molar mass distribution.

Although a further increase of the esteracetal fraction is likely impeded by the solidification of the reaction mixture, the organobase‐catalyzed ROP presented here opens an avenue for new types of degradable polymers. Our future research will concentrate on exploring other hemiacetal ester monomers [17, 25], as well as on exploiting the block copolymers for application as drug carriers with degradable hydrophobic core and “stealth” polymer shells.

## Conflicts of Interest

The authors declare no conflicts of interest.

## Supporting information




**Supporting File 1**: marc202500273‐sup‐0001‐SuppMat.docx.

## Data Availability

The data that support the findings of this study are available in the supplementary material of this article.
