# Supplementary material for: Polyesteracetals via TBD Catalyzed Ring‐Opening Polymerization
Source: Macromol Rapid Commun. 2025 Jul 7;46(18):e00273. doi: 10.1002/marc.202500273 (PMC12447686; doi:10.1002/marc.202500273)
Supplement: Supplementary file 1 — Supporting File 1: marc202500273‐sup‐0001‐SuppMat.docx. [file MARC-46-e00273-s001.docx]

**Supporting Information**

for Macromol. Rapid Commun.

Polyesteracetals *via* TBD catalyzed ring-opening polymerization

Jakob Meyer, Dr. Natalie E. Göppert, Leanne M. Stafast, Dr. Christine Weber, Prof. Dr. Ulrich S. Schubert

**Experimental section**

**Materials**

All chemicals and solvents were purchased from commercial suppliers and used without further purification unless noted otherwise. 2-Methyldihydrofuran-3-one (MDF, >98.0%) was obtained from TCI. *meta*-Chloroperoxybenzoic acid, benzyl alcohol (BnOH, 99.8%), 1,5,7-triazabicyclo[4.4.0]dec-5-ene (TBD, 98%), 1-[3,5-bis(trifluoromethyl)phenyl]-3-[(1*R*,2*R*)-(-)-2-(dimethylamino)cyclohexyl]thiourea (Takemoto’s catalyst, 98%), benzoic acid (PhCOOH, ≥99.5%), pyrene-1-methanol (98.0%), poly(ethylene glycol) (*M*_n_ = 1000 g mol^–1^, PEG_1k_), poly(ethylene glycol) methyl ether (*M*_n_ = 2000 g mol^–1^, PEG_2k_), 0.5 M NaOMe in MeOH and methyl tosylate (MeOTs, 98%) were obtained from Sigma Aldrich. The synthesis of poly(2-ethyl-2-oxazoline) (*M*_n_ = 2000 g mol^–1^, PEtOx) is described in Stafast *et al*..^[1]^ 1-(3,5-Bis(trifluoromethyl)-phenyl)-3-cyclohexylthiourea (TU) was synthesized according to Pratt *et al*..^[2]^ 2-*n*-Nonyl-2-oxazoline (NonOx) and 2-*iso*-propyl-2-oxazoline (*i*PrOx) were synthesized according to published protocols^[3]^ and subsequently dried over calcium hydride, distilled under reduced pressure, and stored under argon atmosphere. MeOTs was distilled under reduced pressure prior to usage.

Methanol (MeOH), acetonitrile (CH_3_CN), tetrahydrofuran (THF) and dichloromethane (CH_2_Cl_2_) were dried in a solvent purification system (SPS, MBRAUN). Extra dry chloroform was obtained from Thermo Scientific (99.9%).

Preparative size exclusion chromatography was performed in THF using BioBeads SX1 from BioRad.

**Instrumentation**

The polymerization of NonOx and *i*PrOx was performed in a microwave synthesizer from Biotage (Initiator+, temperature control, absorption level very high). The glovebox was manufactured by MBRAUN and is equipped with an UNIlab inert gas (nitrogen) purification system, a vacuum pump and high efficiency box filters HEPA H13.

Proton (^1^H) nuclear magnetic resonance (NMR) spectra were measured with a Bruker AC 300 MHz spectrometer. ^13^C-NMR, heteronuclear single quantum correlation spectroscopy (HSQC) NMR and heteronuclear multiple bond correlation (HMBC) spectra were recorded with a Bruker AC 400 MHz spectrometer. All measurements were performed at room temperature using deuterated chloroform as solvent. Chemical shifts (*δ*) are given in parts per million ("ppm"), using the residual, non-deuterated resonance signal of the solvent for chemical shift referencing.

Electrospray ionization mass spectrometry (ESI-MS) was measured on a Bruker TimsTOF mass spectrometer using a calibration standard “ESI-L Low Concentration Tuning Mix” supplied from Agilent.

Matrix-assisted laser desorption ionization time-of-flight mass spectrometry (MALDI-TOF-MS) was performed using a Bruker Daltonics rapifleX MALDI-TOF/TOF system equipped with a smartbeam™ 3D laser (355 nm wavelength). The spectra were measured in positive reflector mode. The instrument was calibrated with an external poly(methyl methacrylate) (PMMA) 2500 g·mol^–1^ standard from PSS. *Trans*-2-[3-(4-*tert*-butylphenyl)-2-methyl-2-propenylidene]malononitrile (DCTB) was used as matrix. Sodium trifluoro acetate (NaTFA) was added as a doping salt.

Size exclusion chromatography (SEC) was measured on a Shimadzu system equipped with a SCL-10A VP system controller, a LC-10AD VP pump and a RID-10A refractive index detector using a solvent mixture containing chloroform, triethylamine, and *iso*-propanol (94/4/2) at a flow rate of 1 mL min^–1^ on a PSS-SDV-linear S 5 μm column (PSS GmbH Mainz, Germany) at 40 °C. The system was calibrated with polystyrene (370 to 128,000 g mol^–1^) standards.

**Synthesis of 2-methyl-1,3-dioxan-4-one (MDO)**

A modified literature procedure was used for the synthesis.^[4]^ The reaction was performed in an argon atmosphere.

*meta*-Chloroperoxybenzoic acid (69.43 g, 0.40 mol, 1.2 eq.) was dissolved in CH_2_Cl_2_ (600 mL). MgSO_4_ was added until the solution was dry. The suspension was subsequently filtered and washed with CH_2_Cl_2_ (100 mL). MDF (25.85 g, 0.26 mol, 1 eq.) was added dropwise to the solution. The mixture was stirred overnight at room temperature. The suspension was subsequently filtered. MgSO_4_ (31.08 g, 0.26 mol, 1 eq.) and Na_2_CO_3_ (64.31 g, 0.61 mol, 2.35 eq.) were added to the solution and stirred vigorously overnight. The solid was filtered off and washed with CH_2_Cl_2_. The organic solvent was evaporated at reduced pressure yielding 19.6 g of crude product. The crude product was dried with CaH_2_ overnight and distilled under reduced pressure (*p* = 0.8 mbar, *T* = 60 °C).

**Yield:** 16.9 g (55%) of a colorless liquid

**Elemental analysis:** Calculation: C, 51.72, H, 6.94

Experimental: C, 51.25, H, 6.92

**^1^H-NMR** (300 MHz, CDCl_3_): *δ* = 1.52 (d, *J* = 5.12 Hz, 3H, CH-C*H*_3_), 1.67 (*H*_2_O), 2.58-2.88 (m, 2H, O=C-C*H*_2_), 3.91-4.27 (ddd, *J* = 11.42, 8.20, 2.40 Hz, 2H, O-C*H*_2_, 5.44 (q, 1H, *J =* 5.12 Hz, C*H*-CH_3_), 7,28 (s, CHCl_3_) ppm.

**^13^C-NMR** (75 MHz, CDCl_3_): *δ* = 21.1 (*C*H_3_), 29.9 (O=C-*C*H_2_), 63.3 (O-*C*H_2_), 77.0 (*C*HCl_3_), 101.2 (O-*C*H), 167.4 (O=*C*) ppm.


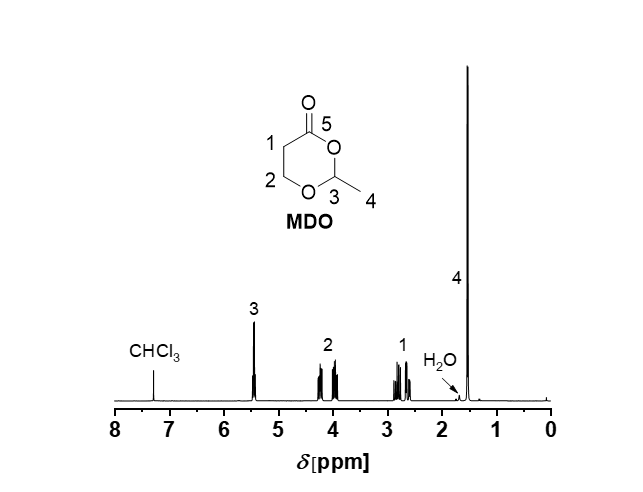


Figure S1: ^1^H-NMR spectrum of MDO (300 MHz, CDCl_3_).

Figure S2: ^13^C-NMR spectrum of MDO (75 MHz, CDCl_3_).


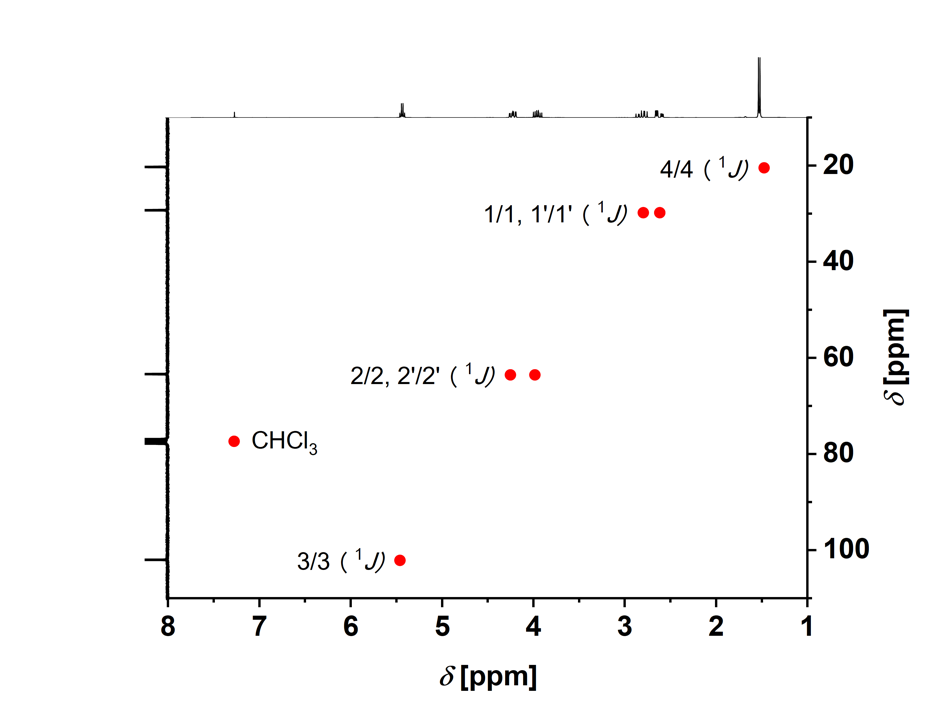


Figure S3: HSQC-NMR spectrum of MDO (400 MHz, CDCl_3_).


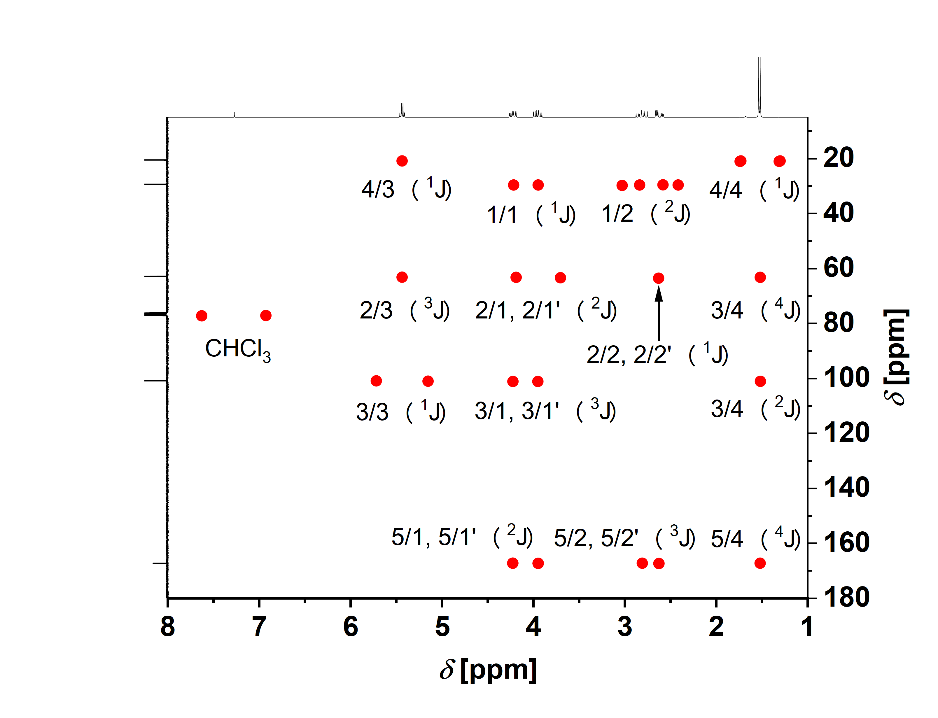


Figure S4: HMBC-NMR spectrum of MDO (400 MHz, CDCl_3_).


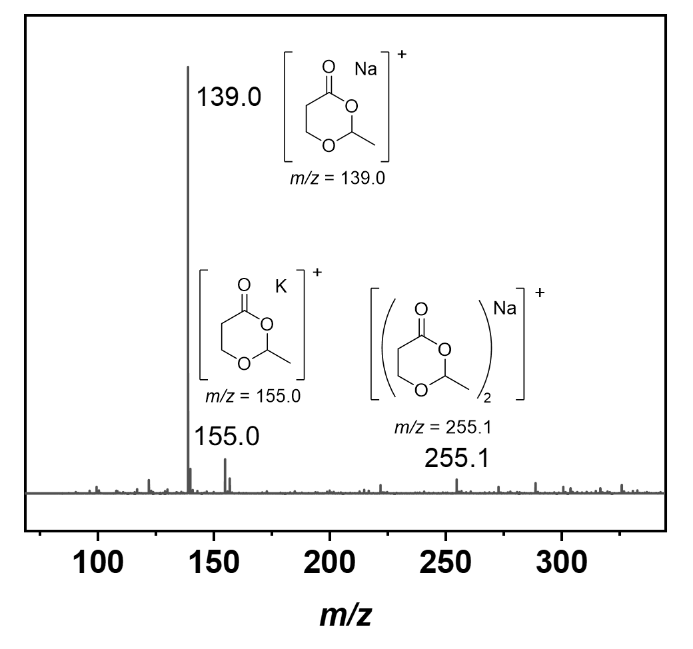


Figure S5: ESI-TOF mass spectrum of MDO.

**General procedure for the polymerization of MDO**

The following general procedure was applied for all polymerizations involving the monomer **MDO**.

The components for the reaction mixture were prepared in a glove box. **MDO** in CHCl_3_ and a stock solution containing the initiator and catalyst in CHCl_3_ were removed from the glove box and adjusted to the desired reaction temperature. 100 μL of the stock solution were added to the monomer solution to give the desired ratio of [M]/[BnOH]/[TBD]. After the desired reaction time, the reaction was quenched with a 20-fold excess of benzoic acid. An aliquot was taken to calculate the conversion by means of ^1^H-NMR spectroscopy. Details are specified in **Table S1**.

For polymers that were analyzed further, the reaction mixture was purified by chromatography on a BioBeads SX1 column in THF. The fractions containing the polymer were collected and the solvent was evaporated under reduced pressure.

Table S1: Polymerization of MDO with BnOH at different parameters.

| **Catalyst** | **[M]/[I]/[cat]/[base]** | **n (M/I/cat/base) [mmol]** | **m (M/I/cat/base) [mg]** | **Solvent [mL]** | ***c* [M]^a)^** | ***T*** | **Conversion [%]^b)^** | **PEA:PE^b)^** |
| --- | --- | --- | --- | --- | --- | --- | --- | --- |
| TU/DBU | 20:1:5:1 | 1.7/0.86/0.43/0.086 | 200/9.31/159.48/13.11 | CHCl_3_, 2.46 | 0.7 | RT | 100 (3 h) | 0:100 |
| TU/DBU | 20:1:5:1 | 1.7/0.86/0.43/0.086 | 200/9.31/159.48/13.11 | CHCl_3_, 0.17 | 10 | 5 °C | 97 (2 h) | 10:90 |
| TU/DBU | 20:1:1:1 | 1.7/0.86/0.086/0.086 | 200/9.31/13.11/31.90 | CHCl_3_, 0.17 | 10 | 5 °C | 96 (2 h) | 13:87 |
| Takemoto | 20:1:1/– | 1.7/0.086/0.086/– | 200/9.31/35.60/– | CHCl_3_, 2.46 | 0.7 | RT | < 5 (24 h) | 0:100 |
| TBD | 20:1:1/– | 1.7/0.86/0.086/– | 200/9.31/12.00/– | CH_2_Cl_2_, 2.46 | 0.7 | RT | 51.6 (3 h) | 0:100 |
| TBD | 20:1:1/– | 1.7/0.86/0.086/– | 200/9.31/12.00/– | CHCl_3_, 0.17 | 10 | RT | 77.4 (55 min) | 19:81 |
| TBD | 20:1:1/– | 1.7/0.86/0.086/– | 200/9.31/12.00/– | CHCl_3_, 0.17 | 10 | 5 °C | 68 (5 h) | 33:67 |
| TBD | 20:1:1/– | 1.7/0.86/0.086/– | 200/9.31/12.00/– | CHCl_3_, 0.17 | 10 | –20 °C | 94 (20 min) | 37:63 |
| TBD | 20:1:1/– | 1.7/0.86/0.086/– | 200/9.31/12.00/– | CHCl_3_, 0.09 | 20 | –24 °C | 54 (20 min) | 64:36 |
| TBD | 20:1:1/– | 1.7/0.86/0.086/– | 200/9.31/12.00/– | CHCl_3_, 0.09 | 20 | –35 °C | 64 (6 h) | 50:50 |
| TBD | 20:1:1/– | 1.7/0.86/0.086/– | 200/9.31/12.00/– | CHCl_3_, 0.09 | 20 | –36 °C | 94 (100 min) | 43:57 |
| TBD | 20:1:1/– | 1.7/0.86/0.086/– | 200/9.31/12.00/– | CHCl_3_, 0.09 | 20 | –42 °C | 56 (2 h) | 55:45 |
| TBD | 20:1:0.2/– | 1.7/0.86/0.017/– | 200/9.31/2.40/– | CH_2_Cl_2_, 2.46 | 0.7 | RT | 30.8 (3 h) | 0:100 |
| TBD | 20:1:0.02/– | 1.7/0.86/0.0017/– | 200/9.31/0.24/– | CHCl_3_, 2.46 | 0.7 | RT | 0 (19.5 h) | 0:100 |
| TBD | 100:1:1/– | 1.7/0.017/0.017/– | 200/1.86/2.4/– | CHCl_3_, 0.17 | 10 | RT | 12 (53 min) | 10:90 |
| TBD | 100:1:0.5/– | 1.7/0.017/0.0086/– | 200/1.86/1.20/– | THF, 2.46 | 0.7 | RT | 7.8 (20 h) | 0:100 |
| TBD | 100:1:1/– | 1.7/0.017/0.017/– | 200/1.86/2.40/– | Toluene, 2.46 | 0.7 | 60 °C | 22.5 (53 min) | 0:100 |
| TBD | 100/1/1 | 1.7/0.017/0.017/– | 200/1.86/2.4/– | CHCl_3_, 0.04 | 20 | –35 °C | 39.8 (80 min) | 18:82 |
| TBD | 100/1/5 | 1.7/0.017/0.086/– | 200/1.86/12.0/– | CHCl_3_, 0.04 | 20 | –35 °C | 56.9 (130 min) | 34:66 |

1. Concentration neglects the volume of **MDO**. b) Estimated by means of ^1^H-NMR spectroscopy.

**Kinetic studies of the polymerization of MDO with benzyl alcohol as initiator**

The kinetic studies were carried out as described in the general procedure using BnOH as initiator. Corresponding to a [M]/[I][cat] ratio of [100]/[1][/5], **MDO** (200 mg, 1.72 mmol), BnOH (1.86 mg, 0.017 mmol) and TBD (12 mg, 0.086 mmol) were used. Samples were taken after 5 min, 10 min, 20 min, 40 min, 100 min and 160 min, respectively, and analyzed by means of ^1^H-NMR spectroscopy and SEC.

**Synthesis of BnPMDO**

The reaction was carried out as described in the general procedure using **MDO** (500 mg, 4.31 mmol), BnOH (4.66 mg, 0.043 mmol), TBD (59.94 mg, 0.43 mmol) and CHCl_3_ (285 μL).

**Conversion:** 46%

**Isolated yield:** 137 mg (27.4%)

**SEC:** *M*_n_ = 1900 g mol^–1^

*Đ* = 1.37

**^1^H-NMR** (300 MHz, CDCl_3_): *δ* = 1.40 (d, 3H, *J* = 5.03 Hz, C*H*_3_), 2.57-2.72 (m, 4H, O=C-C*H*_2_), 3.71-3.95 (m, 2H, CH-O-C*H*_2_), 4.35-4.43 (m, 2H, O-C*H*_2_), 5.17 (s, 2H, Bn-C*H*_2_), 5.94-5.99 (q, 1H, C*H*), 7.27 (s, 1H, C*H*Cl_3_), 7.36 (s, 5H, Ar-*H*) ppm.

**Synthesis of PyPMDO**

The reaction was carried out as described in the general procedure using **MDO** (500 mg, 4.31 mmol), 1-pyrenemethanol (10.00 mg, 0.043 mmol), TBD (59.94 mg, 0.43 mmol) and CHCl_3_ (285 μL).

**Conversion:** 25%

**Isolated yield:** 62 mg (12.4%)

**SEC:** *M*_n_ = 1500 g mol^–1^

*Đ* = 1.15

**^1^H-NMR** (300 MHz, CDCl_3_): *δ* = 1.40 (d, 3H, *J* = 5.02 Hz, C*H*_3_), 1.68 (*H*_2_O), 2.50-2.72 (m, 4H, O=C-C*H*_2_), 3.71-3.95 (m, 2H, CH-O-C*H*_2_), 4.35-4.43 (m, 2H, O-C*H*_2_), 5.91 (s, 2H, Py-C*H*_2_), 5.94-5.99 (q, 1H, C*H*), 7.27 (s, 1H, C*H*Cl_3_), 8.02-8.32 (m, 9H, Py-*H*) ppm.

**Synthesis of macroinitiators**

**Polymerization of 2-*n*-nonyl-2-oxazoline with MeTos as initiator**

The polymerization was adapted from the procedure described in the literature for PEtOx.^[5]^

The reaction solution was prepared in a glove box. NonOx (1.8 g, 9.12 mmol, 10 eq.) and MeTos (138 μL, 0.091 mmol, 1 eq.) were dissolved in CH_2_Cl_2_ (2.62 mL). The vial was capped and transferred out of the glove box. The solution was then heated in a microwave synthesizer to 140 °C for 3:30 minutes. Subsequently, the solution was quenched with acetic acid (80 µL, 1.37 mmol, 1.5 eq.) and NEt_3_ (250 µL, 1.82 mmol, 2 eq.). The mixture was stirred at 50 °C overnight. An aliquot was taken to determine the monomer conversion by means of ^1^H-NMR spectroscopy (quantitative). The solution was diluted using CH_2_Cl_2_ (30 mL), followed by washing with sat. aq. NaHCO_3_ (2×45 mL) and brine (1×45 mL). The organic phase was dried over MgSO_4_ and filtered. The solvent was removed using a rotary evaporator and a white solid was obtained, which was used directly for the synthesis of **PNonOx**.

**SEC:** *M*_n_ = 2900 g mol^–1^

*Đ* = 1.14

**^1^H-NMR** (300 MHz, CDCl_3_): *δ* = 0.87 (br t, *J* = 8.94 Hz, 3H, C*H*_3_), 1.26 (br s, 12H, 6×C*H*_2_), 1.58 (br s, 2H, O=C-CH_2_-C*H*_2_), 2.00-2.03 (m, 3H, O=C-C*H*_3_), 2.21-2.29 (m, 2H, O=C-C*H*_2_), 2.94-3.02 (m, 3H, C*H*_3_-N), 3.43-3.78 (m, 4H, N-C*H*_2_-C*H*_2_), 4.15 (br s, 2H, O-C*H*_2_), 5.30 (C*H*_2_Cl_2_) ppm.

**Synthesis of PNonOx**

MeOH (12 mL) was added to **PNonOx-OAc** (1.5 g, 0.76 mmol). An additional 5 mL of dry CHCl_3_ was added to dissolve the **PNonOx-OAc**. Then, 0.5 M MeONa (140 µL, 0.07 mmol) in MeOH was added to the solution, followed by stirring overnight. The solvent was subsequently removed and CH_2_Cl_2_ (100 mL) was added. The organic phase was washed with saturated aq. NaHCO_3_ (1×80 mL) solution and brine (1×80 mL). The organic phase was dried with MgSO_4_ and filtered. The solvent was then removed under reduced pressure.

**Yield:** 945 mg (48%)

**SEC:** *M*_n_ = 2800 g mol^–1^

*Đ* = 1.13

**^1^H-NMR** (300 MHz, CDCl_3_): *δ* = 0.87 (br t, *J* = 8.94 Hz, 3H, C*H*_3_), 1.26 (br s, 12H, 6×C*H*_2_), 1.58 (br s, 2H, O=C-CH_2_-C*H*_2_), 1.94 (*H*_2_O), 2.21-2.29 (m, 2H, O=C-C*H*_2_), 2.94-3.02 (m, 3H, C*H*_3_-N), 3.43-3.78 (m, 4H, N-C*H*_2_-C*H*_2_), 7.27 (s, 1H, C*H*Cl_3_) ppm.

**Kinetic studies of the cationic ring-opening polymerization (CROP) of 2-*iso*-propyl-2-oxazoline with MeTos as initiator**

The polymerization was carried out in microwave vials which were prepared in a glove box.

A stock solution containing *i*PrOx (4 g, 35.35 mmol, 20 eq.), MeTos (266.73 μL, 1.77 mmol, 1 eq.) and CH_3_CN (13.41 mL) was prepared and divided into 12 aliquots of 0.9 mL each. The vials were capped and transferred out of the glove box.

Polymerization was carried out in the microwave synthesizer at 140 °C with the following reaction times: 1 s, 10 s, 20 s, 30 s, 1 min, 1.5 min, 2 min, 3 min, 5 min, 8 min, 11 min, 14 min, 17 min and 20.8 min, respectively. Aliquots were taken for analysis by means of ^1^H-NMR spectroscopy and SEC.

**Polymerization of 2-*iso*-proyl-2-oxazoline with MeTos as initiator**

The polymerization was carried out in a microwave vial which was prepared in a glove box.

*i*PrOx (927 mg, 8,20 mmol, 20 eq.) and MeTos (62 μL, 0.41 mmol, 1 eq.) were dissolved in CH_3_CN (2.47 mL). The vial was capped and transferred out of the glove box. The solution was then heated to 140 °C for 25 min using a microwave synthesizer to achieve 99.9% conversion according to the kinetic studies. Subsequently, the solution was quenched with acetic acid (35 µL, 0.62 mmol, 1.5 eq.) and NEt_3_ (120 µL, 0.82 mmol, 2 eq.). The mixture was stirred at 50 °C overnight. An aliquot was taken to determine the conversion by means of ^1^H-NMR spectroscopy (quantitative). The resulting solution was diluted using CH_2_Cl_2_ (30 mL), followed by washing with sat. aq. NaHCO_3_ (2×45 mL) and brine (1×45 mL). The organic phase was dried over MgSO_4_ and filtered. The solvent was removed using a rotary evaporator and a white solid was obtained.

**Yield:** 407 mg (42%)

**SEC:** *M*_n_ = 2800 g mol^–1^

*Đ* = 1.14

**^1^H-NMR** (300 MHz, CDCl_3_): *δ* = 1.13 (br s, 6H, 2×C*H*_3_), 1.61 (*H*_2_O), 2.09 (s, 3H, O=C-CH_3_), 2.68-2.95 (br d, 1H, C*H*), 3.09 (m, 3H, C*H*_3_-N), 3.47-3.83 (m, C*H*_2_-C*H*_2_), 4.21 (br s, 2H, O-C*H*_2_), 5.32 (CH_2_Cl_2_), 7.27 (s, 1H, C*H*Cl_3_) ppm.

**Synthesis of P*i*PrOx**

MeOH (5 mL) was added to **P*i*PrOx-OAc** (407 mg). Subsequently, 0.5 M MeONa (40 µL, 0.02 mmol) in MeOH was added, followed by stirring overnight. The solvent was then removed, and CH_2_Cl_2_ (50 mL) was added. The organic phase was washed with sat. aq. NaHCO_3_ (1×35 mL) solution and brine (1×35 mL). The organic phase was dried with MgSO_4_ and filtered. The solvent was removed under reduced pressure and a white solid was obtained.

**Yield:** 353 mg (88%)

**SEC:** *M*_n_ = 2900 g mol^–1^

*Đ* = 1.08

**^1^H-NMR** (300 MHz, CDCl_3_): *δ* = 1.13 (br s, 6H, 2×C*H*_3_), 1.61 (*H*_2_O), 2.68-2.95 (br d, 1H, C*H*), 3.09 (m, 3H, C*H*_3_-N), 3.47-3.83 (m, C*H*_2_-C*H*_2_), 5.32 (CH_2_Cl_2_), 7.27 (s, 1H, C*H*Cl_3_) ppm.

**Synthesis of block copolymers**

**Synthesis of PMDO-PEG-PMDO**

The reaction was carried out at –35 °C, as described in the general procedure using **MDO** (500 mg, 4.31 mmol), PEG_1k_ (43.06 mg, 0.043 mmol), TBD (59.94 mg, 0.43 mmol) and CHCl_3_ (285 μL) yielding **PMDO-PEG-PMDO**.

**Conversion:** 34%

**Isolated yield:** 141 mg (26%)

**SEC:** *M*_n_ = 5600 g mol^–1^

*Đ* = 1.06

**^1^H-NMR** (300 MHz, CDCl_3_): *δ* = 1.40 (d, 3H, *J* = 5.02 Hz, C*H*_3_), 1.63 (*H*_2_O), 2.50-2.72 (m, 4H, O=C-C*H*_2_), 3.66 (s, 4H, C*H*_2_-C*H*_2_ (PEG)) 3.71-3.95 (m, 2H, CH-O-C*H*_2_), 4.35-4.43 (m, 2H, O-C*H*_2_), 5.94-5.99 (q, 1H, C*H*), 7.27 (s, 1H, C*H*Cl_3_) ppm.

**Synthesis of PEG-PMDO**

The reaction was carried out at –35 °C as described in the general procedure using **MDO** (500 mg, 4.31 mmol), PEG_2k_ (86.12 mg, 0.043 mmol), TBD (29.97 mg, 0.22 mmol) and CHCl_3_ (285 μL) yielding **PEG-PMDO**.

**Conversion:** 27%

**Isolated yield:** 110 mg (n.d.)

**SEC:** *M*_n_ = 6400 g mol^–1^

*Đ* = 1.05

**^1^H-NMR** (300 MHz, CDCl_3_): *δ* = 1.40 (m, 3H, C*H*_3_), 1.74 (*H*_2_O), 2.50-2.72 (m, 4H, O=C-C*H*_2_), 3.66 (s, 4H, C*H*_2_-C*H*_2_ (PEG)) 3.71-3.95 (m, 2H, CH-O-C*H*_2_), 4.35-4.43 (m, 2H, O-C*H*_2_), 5.94-5.99 (q, 1H, C*H*), 7.27 (s, 1H, C*H*Cl_3_) ppm.

**Synthesis of PEtOx-PMDO**

The reaction was carried out at –35 °C as described in the general procedure using **MDO** (500 mg, 4.31 mmol), PEtOx (81.82 mg, 0.043 mmol), TBD (59.94 mg, 0.43 mmol) and CHCl_3_ (285 μL).

**Conversion:** 42%

**Isolated yield:** 169 mg (29%)

**SEC:** *M*_n_ = 3800 g mol^–1^

*Đ* = 1.10

**^1^H-NMR** (300 MHz, CDCl_3_): *δ* = 1.14 (br s, 3H, CH*H*_3_ (PEtOx)), 1.40 (m, 3H, C*H*_3_), 1.69 (*H*_2_O), 2.3-2.42 (m, 2H, CH_3_-C*H*_2_ (PEtOx)) 2.50-2.72 (m, 4H, O=C-C*H*_2_), 3.05 (m, 3H, N-C*H*_3_ (PEtOx)), 3.47 (br s, 4H, C*H*_2_-C*H*_2_ (PEtOx)), 3.75-3.95 (m, 2H, CH-O-C*H*_2_), 4.35-4.43 (m, 2H, O-C*H*_2_), 5.94-5.99 (q, 1H, C*H*), 7.27 (s, 1H, C*H*Cl_3_) ppm.

**Synthesis of PNonOx-PMDO**

The reaction was carried out at –35 °C as described in the general procedure using **MDO** (500 mg, 4.31 mmol), **PNonOx** (86.35 mg, 0.043 mmol), TBD (59.94 mg, 0.43 mmol) and CHCl_3_ (428 μL).

**Conversion:** 18%

**Isolated yield:** 116 mg (20%)

**SEC:** *M*_n_ = 3100 g mol^–1^

*Đ* = 1.18

**^1^H-NMR** (300 MHz, CDCl_3_): *δ* = 0.89 (br t, 3H, C*H*_3_ (PNonOx)), 1.28 (br s, 12H, 6×C*H*_2_ (PNonOx)), 1.40 (m, 3H, C*H*_3_), 1.63 (br s, 2H, O=CH_2_-C*H*_2_ (PNonOx)), 2.26-2.34 (m, 2H, O=C-C*H*_2_ (PNonOx)), 2.57-2.70 (m, 4H, O=C-C*H*_2_), 3.04 (m, 3H, N-C*H*_2_ (PNonOx)), 3.45 (br s, 4H, C*H*_2_-C*H*_2_ (PNonOx)), 3.73-3.95 (m, 2H, CH-O-C*H*_2_), 4.35-4.43 (m, 2H, O-C*H*_2_), 5.94-5.99 (q, 1H, C*H*), 7.27 (s, 1H, C*H*Cl_3_) ppm.

**Synthesis of P*i*PrOx-PMDO**

The reaction was carried out at –35 °C as described in the general procedure using **MDO** (500 mg, 4.31 mmol), **P*i*PrOx** (98.82 mg, 0.043 mmol), TBD (59.94 mg, 0.43 mmol) and CHCl_3_ (285 μL).

**Conversion:** 17%

**Isolated yield:**162 mg (27%)

**SEC:** *M*_n_ = 3300 g mol^–1^

*Đ* = 1.09

**^1^H-NMR** (300 MHz, CDCl_3_): *δ* = 1.13 (br s, 6H, C*H*_3_ (P*i*PrOx)), 1.60 (*H*_2_O), 1.40 (m, 3H, C*H*_3_), 1.83 (THF), )), 2.57-2.70 (m, 4H, O=C-C*H*_2_), 2.5-3.06 (m, 1H, O=C*H* (P*i*PrOx), 3.10 (m, 3H, N-C*H*_2_ (P*i*PrOx)), 3.77 (THF), 3.73-3.95 (m, 2H, CH-O-C*H*_2_), 4.35-4.43 (m, 2H, O-C*H*_2_), 5.94-5.99 (m, 1H, C*H*), 7.27 (s, 1H, C*H*Cl_3_) ppm.

**Supplementary Schemes**


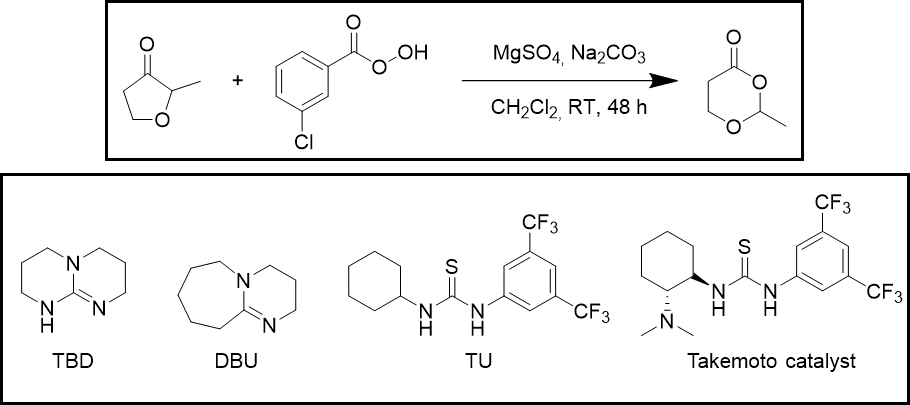


Scheme S1: Top: Schematic representation of the Bayer-Villiger oxidation of 2-methyldihydrofuran-3(2*H*)-one to yield MDO. Bottom: Schematic representation of all catalysts used for the polymerization of MDO.

Scheme S2: Schematic representation of the synthesis of poly(2-oxazoline) macroinitiators.

**Supplementary Figures**


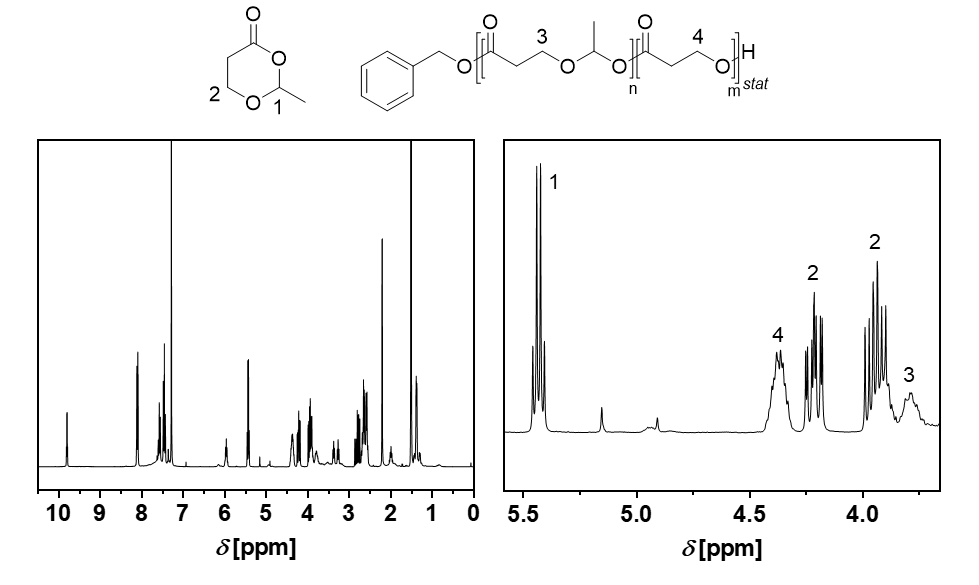


Figure S6: ^1^H-NMR spectrum of the reaction solution of the ring-opening polymerization (ROP) of MDO with BnOH as initiator after 100 min (300 MHz, CDCl_3_). The [MDO]/[BnOH]/[TBD] ratio was [100]/[1]/[5] and the temperature was –35 °C. The assigned signals were used to estimate the monomer conversion and the ratio of ester and esteracetal repeating units in the polymer.

Figure S7: Overlay of the SEC elugrams measured during the kinetic studies of the BnOH initiated ROP of MDO (CHCl_3_, RI detection).


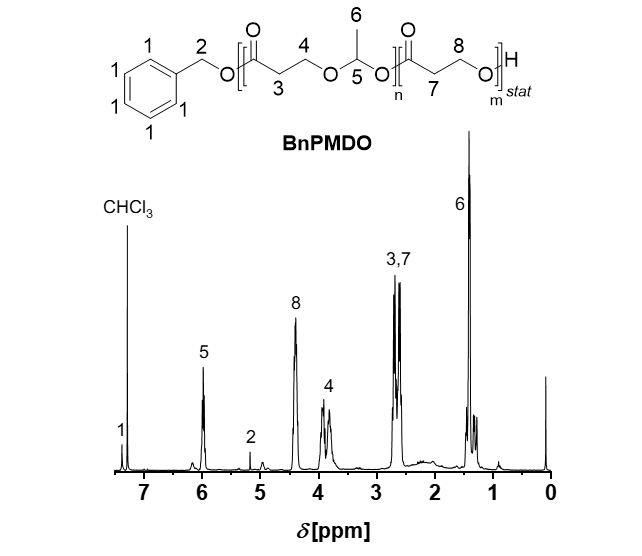


Figure S8: ^1^H-NMR spectrum of BnPMDO (300 MHz, CDCl_3_).

Figure S9: SEC elugrams of BnPMDO after the reaction and after column chromatography (CHCl_3_, RI detection).


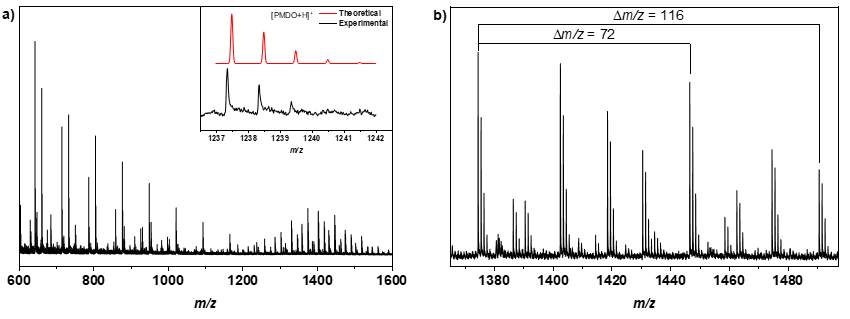


Figure S10: a) MALDI-TOF mass spectrum of the purified BnPMDO (DCTB, NaTFA). The inseted graph shows an overlay of the measured (black) and calculated (red) isotopic patterns of the found species [BnPMDO + H]^+^. b) Zoom in the MALDI-TOF mass spectrum showing the *m/z* differences associated with the repeating units in the polymer.


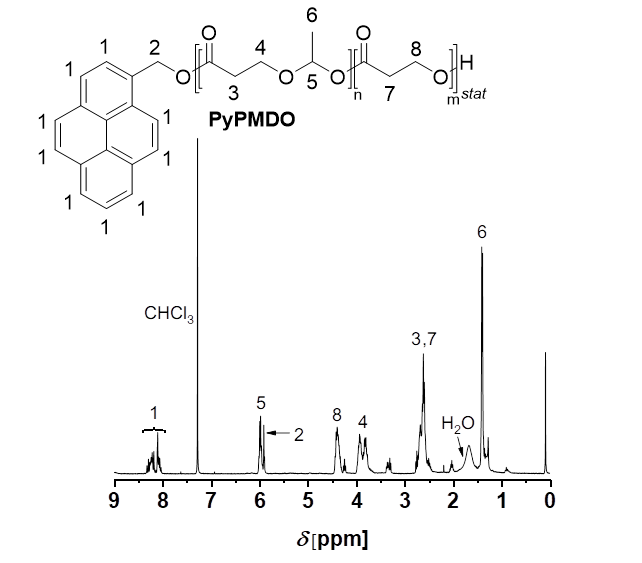


Figure S11: ^1^H-NMR spectrum of PyPMDO (300 MHz, CDCl_3_).

Figure S12: SEC elugrams of PyPMDO after column chromatography (CHCl_3_, UV/Vis detection at 300 nm and RI detection).


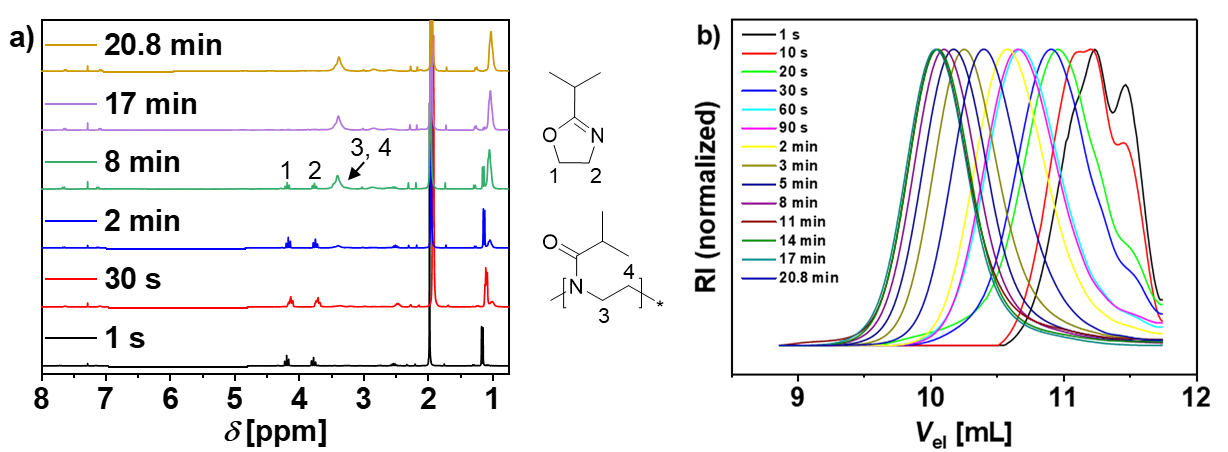


Figure S13: a) ^1^H-NMR spectra measured during the kinetic studies of the CROP of *i*PrOx, showing every third and the last aliquot (300 MHz, CDCl_3_). b) Overlay of the SEC elugrams measured during the kinetic studies of the CROP of *i*PrOx (CHCl_3_, RI detection).


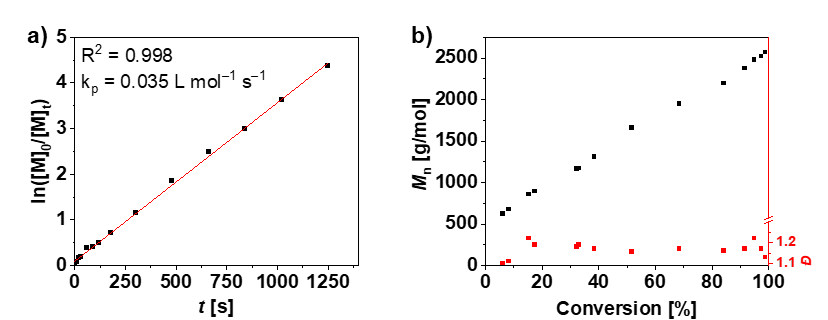


Figure S14: a) First-order kinetic plot of the CROP of *i*PrOx. Linear regression was performed according to ln([M]_0_/[M]_t_) = k_p_ [I]_0_ t. b) *M*_n_ and dispersity *versus* conversion for P*i*PrOx. *M*_n_ and *Đ* were determined by SEC calibrated to a polystyrene standard.


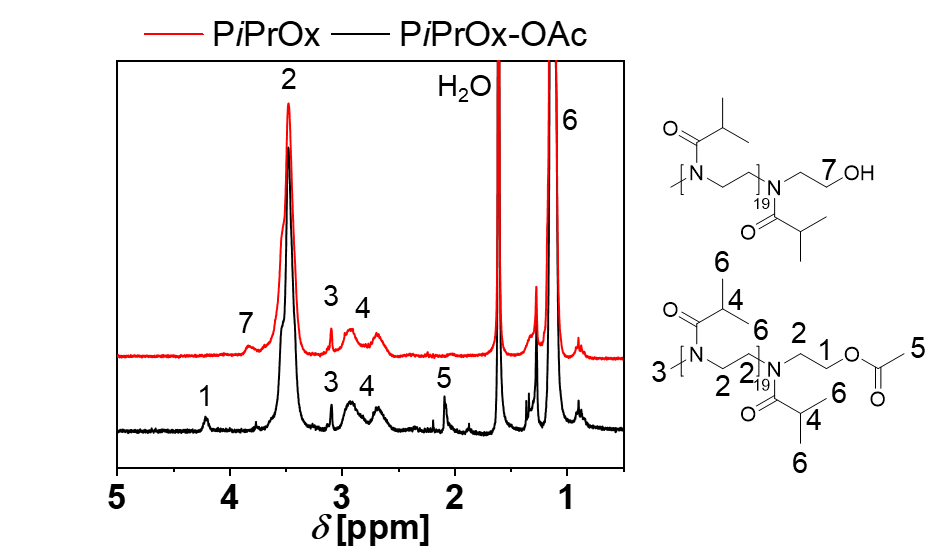


Figure S15: ^1^H-NMR spectra of P*i*PrOx-OAc and P*i*PrOx (300 MHz, CDCl_3_).


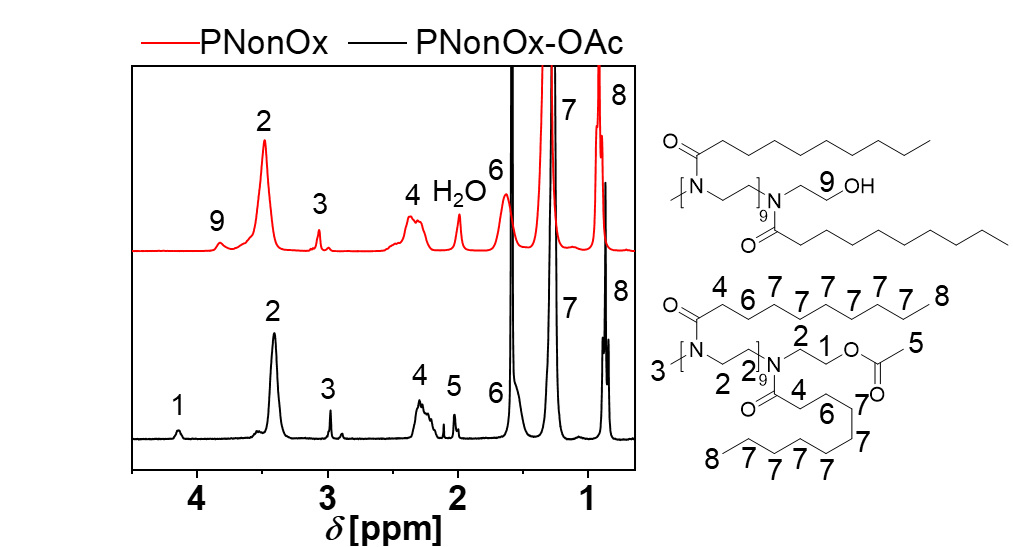


Figure S16: ^1^H-NMR spectra of PNonOx-OAc and PNonOx (300 MHz, CDCl_3_).


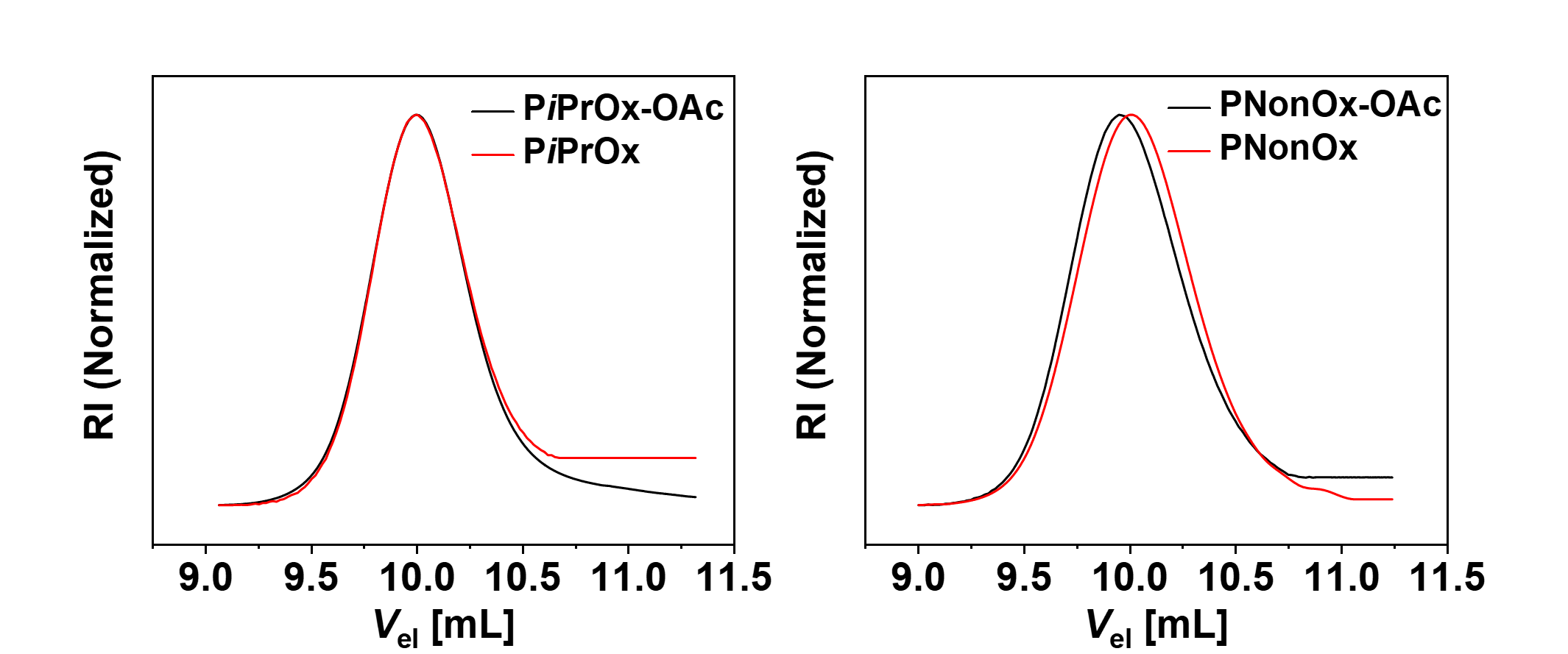


Figure S17: SEC elugrams of P*i*PrOx (left) and PNonOx (right) after quenching with HOAc and after transesterification (CHCl_3_, RI detection).


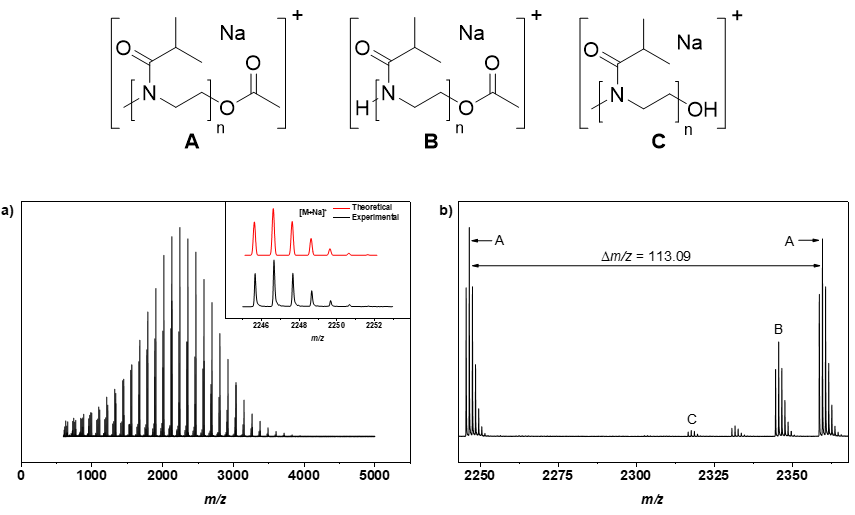


Figure S18: a) MALDI-TOF mass spectrum of the purified P*i*PrOx-OAc (DCTB, NaTFA). The inseted graph shows an overlay of the measured (black) and calculated (red) isotopic patterns of the most prominent species A. b) Zoom into the MALDI-TOF mass spectrum and assignment of the detected species (A, B, C).


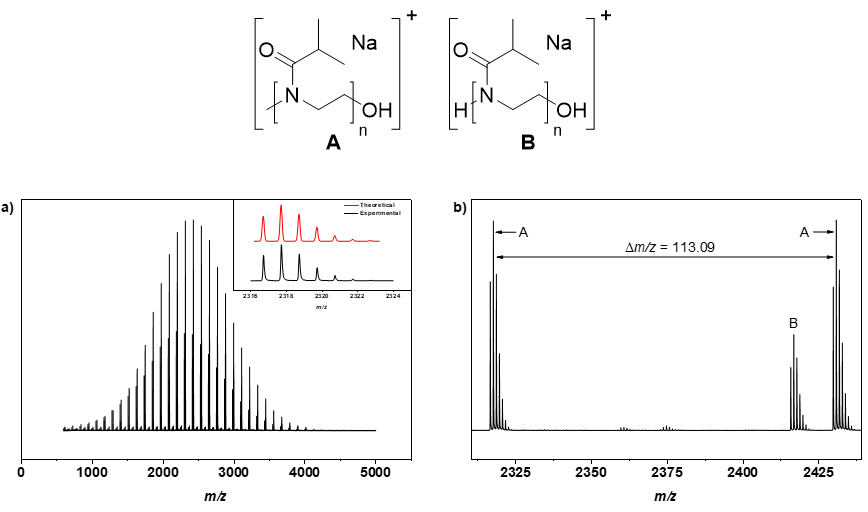


Figure S19: a) MALDI-TOF mass spectrum of the purified P*i*PrOx (DCTB, NaTFA). The inseted graph shows an overlay of the measured (black) and calculated (red) isotopic patterns of the most prominent species A. b) Zoom into the MALDI-TOF mass spectrum and assignment of the detected species (A, B).


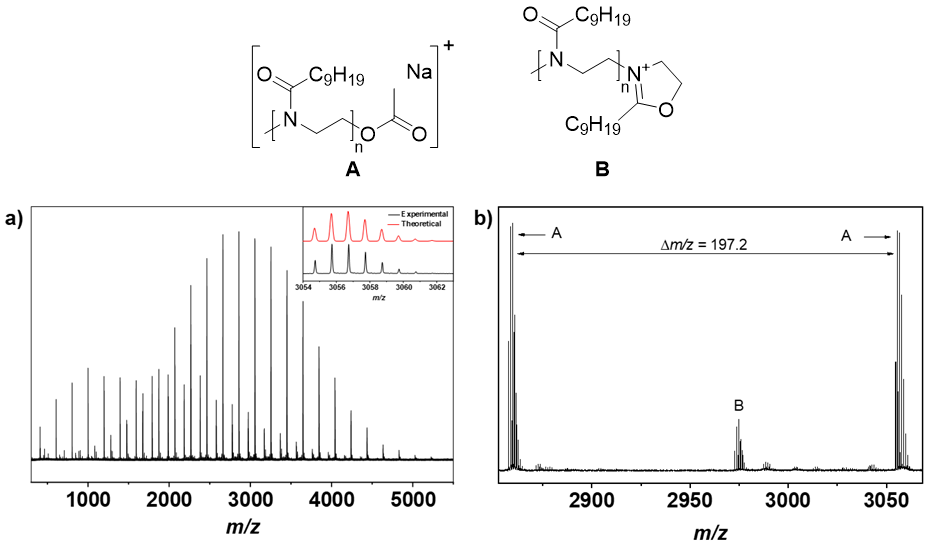


Figure S20: a) MALDI-TOF mass spectrum of the purified PNonOx-OAc (DCTB, NaTFA). The inseted graph shows an overlay of the measured (black) and calculated (red) isotopic pattern of the most prominent species (A). b) Zoom into the MALDI-TOF mass spectrum and assignment of the detected species A and B.


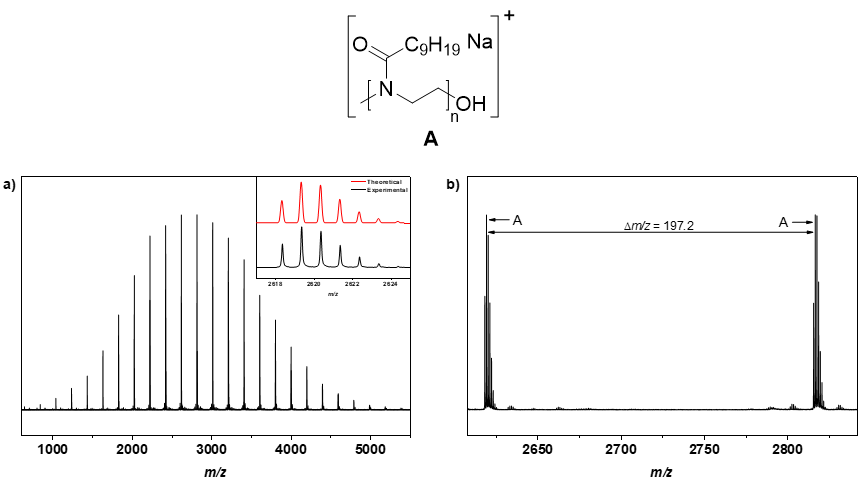


Figure S21: a) MALDI-TOF mass spectrum of the purified PNonOx (DCTB, NaTFA). The inseted graph shows an overlay of the measured (black) and calculated (red) isotopic pattern of the most prominent species. b) Zoom into the MALDI-TOF mass spectrum and assignment of the detected species A.


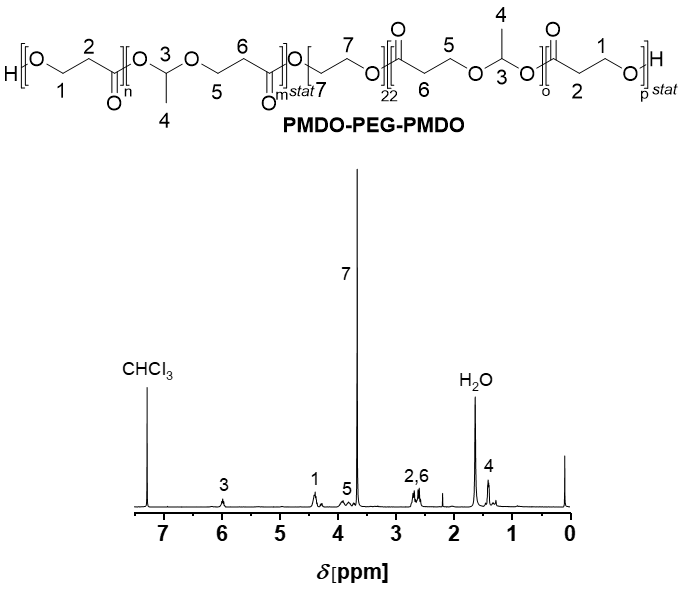


Figure S22: ^1^H-NMR spectrum of PMDO-PEG-PMDO (300 MHz, CDCl_3_).


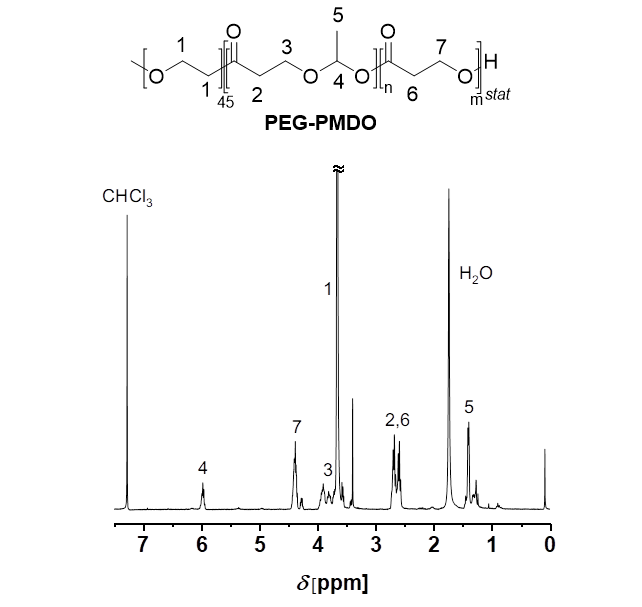


Figure S23: ^1^H-NMR spectrum of PEG-PMDO (300 MHz, CDCl_3_).


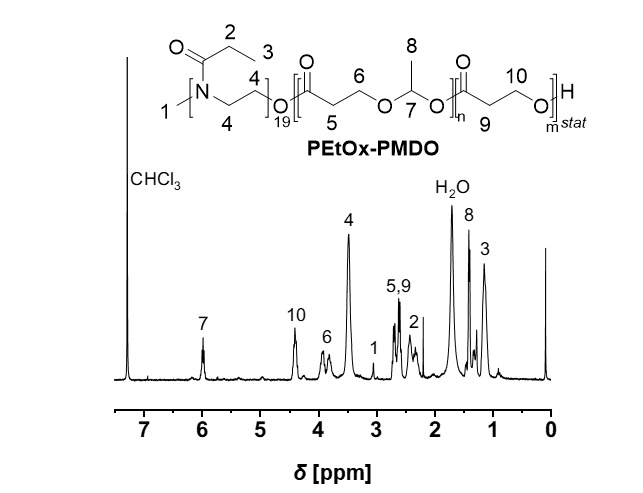


Figure S24: ^1^H-NMR spectrum of PEtOx-PMDO (300 MHz, CDCl_3_).


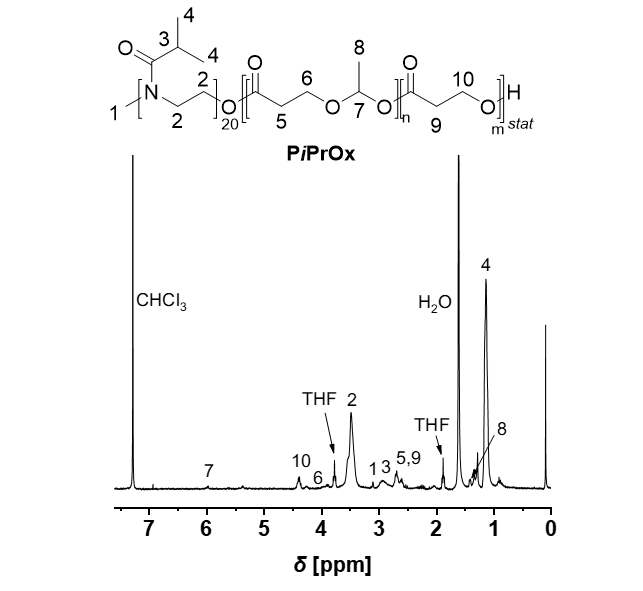


Figure S25: ^1^H-NMR spectrum of P*i*PrOx-PMDO (300 MHz, CDCl_3_).


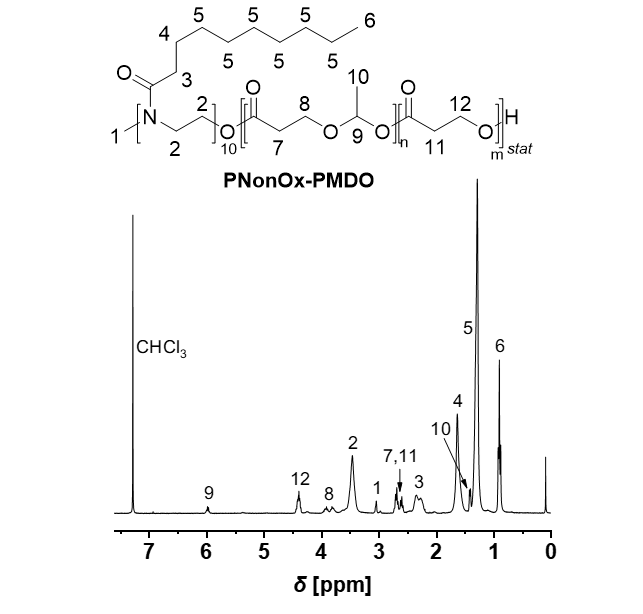


Figure S26: ^1^H-NMR spectrum of PNonOx-PMDO (300 MHz, CDCl_3_).

**References**

[1] L. M. Stafast, M. Swieczkowski, P. Poudel, N. Engel, C. Yin, K. Scheuer, C. Weber, F. H. Schacher, K. D. Jandt, U. S. Schubert, *Eur. Polym. J.* **2024**, 113545.

[2] R. C. Pratt, B. G. G. Lohmeijer, D. A. Long, P. N. P. Lundberg, A. P. Dove, H. Li, C. G. Wade, R. M. Waymouth, J. L. Hedrick, *Macromolecules* **2006**, *39*, 7863-7871.

[3] K. Kempe, M. Lobert, R. Hoogenboom, U. S. Schubert, *J. Comb. Chem.* **2009**, *11*, 274-280.

[4] A. E. Neitzel, M. A. Petersen, E. Kokkoli, M. A. Hillmyer, *ACS Macro Lett.* **2014**, *3*, 1156-1160.

[5] M. Dirauf, A. Erlebach, C. Weber, S. Hoeppener, J. R. Buchheim, M. Sierka, U. S. Schubert, *Macromolecules* **2020**, *53*, 3580-3590.
